# Supplementary material for: Gamified Adaptive Approach Bias Modification in Individuals With Methamphetamine Use History From Communities in Sichuan: Pilot Randomized Controlled Trial
Source: JMIR Serious Games. 2025 Mar 10;13:e56978. doi: 10.2196/56978 (PMC11931399; doi:10.2196/56978)
Supplement: Multimedia Appendix 1 [file games-v13-e56978-s001.pdf]

Participant Information

1. **Age**:
    - ☐ Less than 18
    - ☐ 18-25
    - ☐ 26-35
    - ☐ 36-45
    - ☐ 56-60
    - ☐ Above 60
2. **Gender**:
    - ☐ Male
    - ☐ Female
3. **Education Level**:
    - ☐ Elementary school or below
    - ☐ Junior high school
    - ☐ Senior high school
    - ☐ College or above
4. **Marital Status**:
    - ☐ Married
    - ☐ Unmarried
    - ☐ Divorced
5. **Dominant Hand**:
    - ☐ Right
    - ☐ Left

Mental Disorder Diagnosis

6. **Are you diagnosed with any mental disorders other than MUD? Please list them if any**
    - ☐ [____]

Substance Use History

7. **How many years have you used methamphetamine?**

☐ 1-2 years

☐ 3-5 years

☐ 6-10 years

☐ Over 10 years

8. **Have you used any other substances in the past? (Check all that apply)**

☐ Heroin

- [ ] Ketamine
- [ ] None

9. **Do you smoke?**

- [ ] Yes
- [ ] No

10. **Do you consume alcohol?**

- [ ] Yes
- [ ] No

Smartphone Usage

11. **Are you able to fluently operate a smartphone?**

- [ ] Yes
- [ ] No
