# Supplementary material for: Gamified Adaptive Approach Bias Modification in Individuals With Methamphetamine Use History From Communities in Sichuan: Pilot Randomized Controlled Trial
Source: JMIR Serious Games. 2025 Mar 10;13:e56978. doi: 10.2196/56978 (PMC11931399; doi:10.2196/56978)

Description of WonderLab Harbour

WonderLab Harbour is a digital therapeutic smartphone application developed by Adai Technology (Beijing) Co. Ltd.

# Assessments

Upon first logging-in and at the designated assessment time points, the user goes through assessments for craving for healthy lifestyles and drugs on a 10-point scale (Figure A). The images depicted either methamphetamine-related items (such as crystals, powders, or paraphernalia) or representations of healthy living (e.g., wealth, sports, gourmet food, family activities, etc.).

Figure A: baseline asssesments


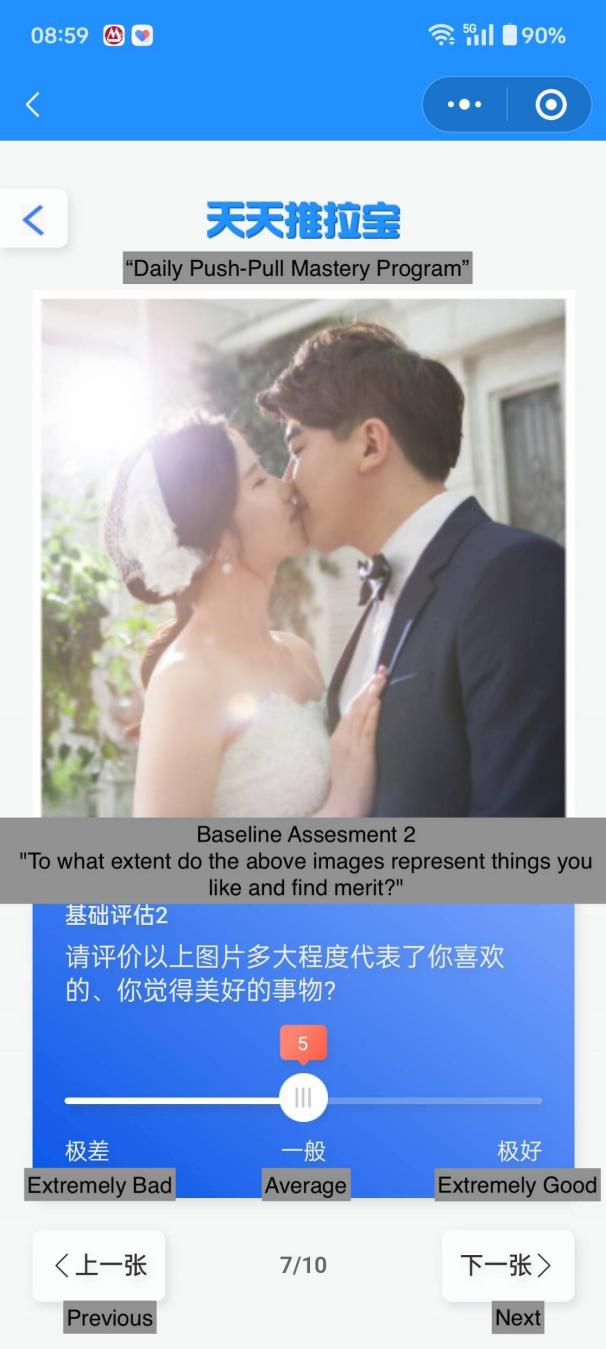

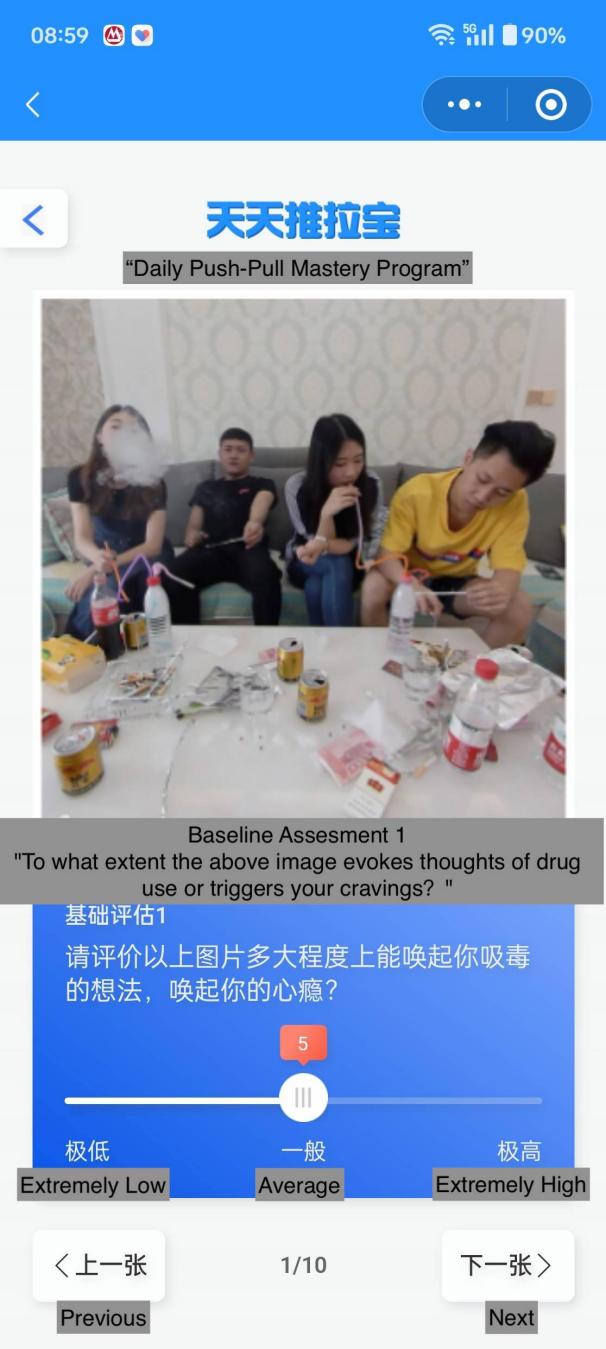


# Instructions and Practice Trials

The users are given instructions and two practice trials (Figures B and C).

Figure B: instructions


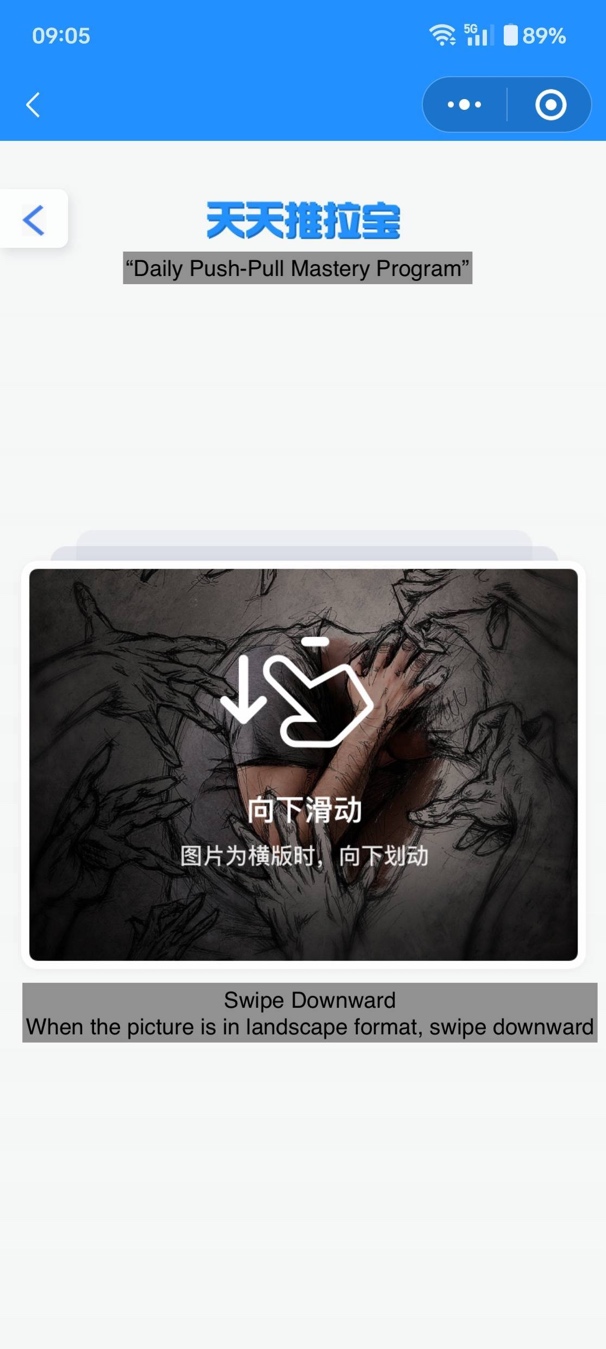

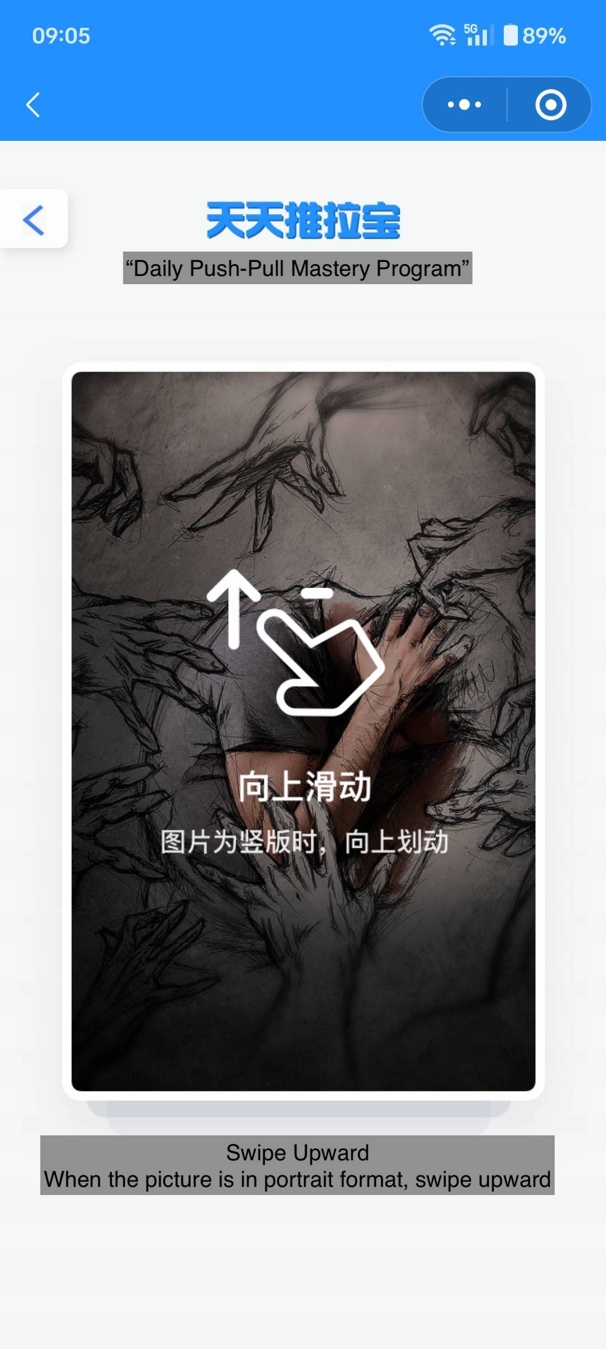


Figure C: practice trials


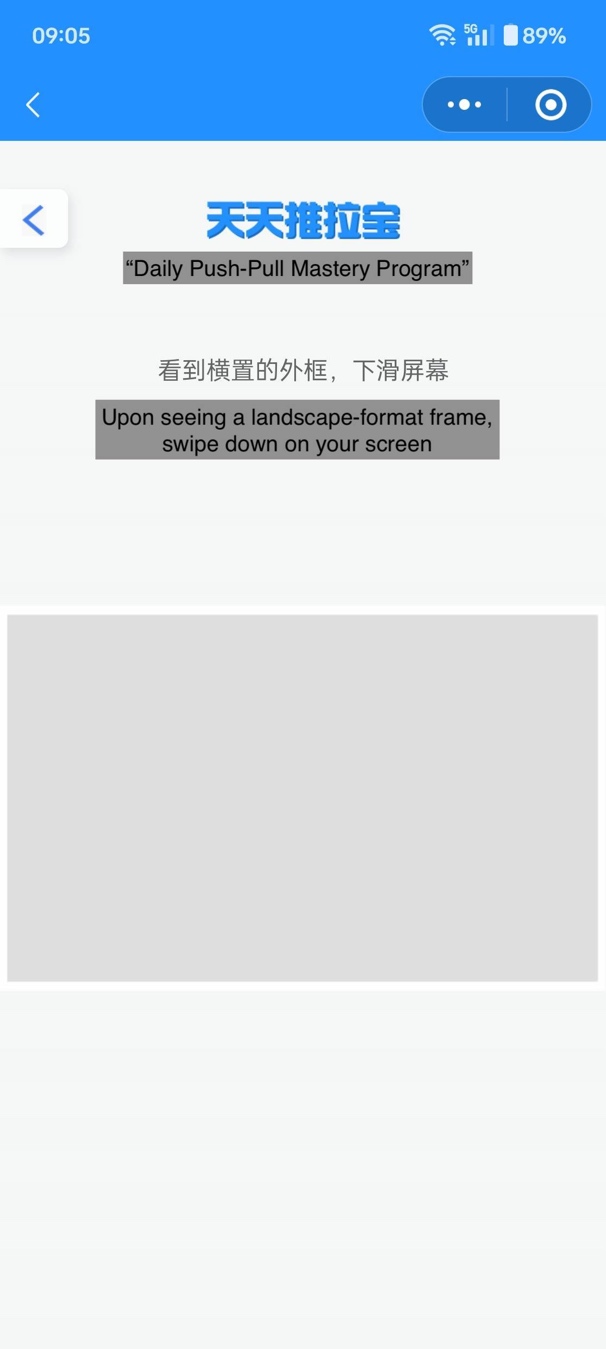

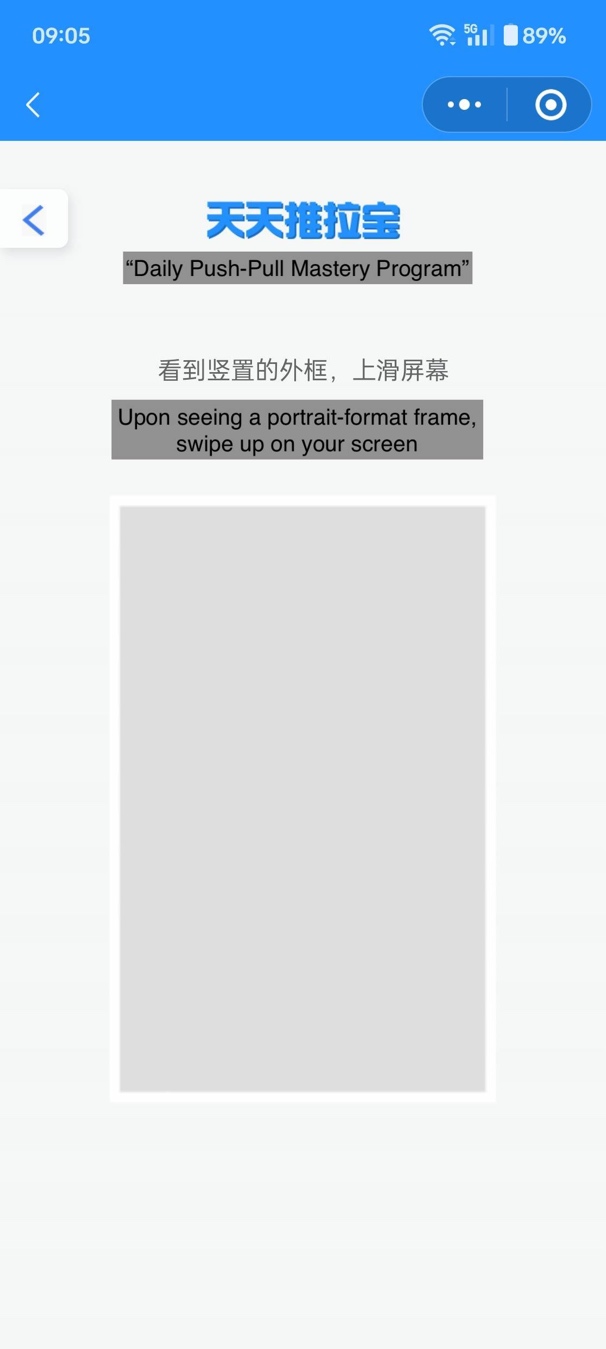


# ApBM and Game Rewards

In eachApBM session, participants were instructed to swipe upward (or downward) when shown images in portrait (or landscape) orientation. After swiping upward (downward), an animation either shrank (grew) to create the effect of distancing (approaching) the object.

Figure D: ApBM and game rewards


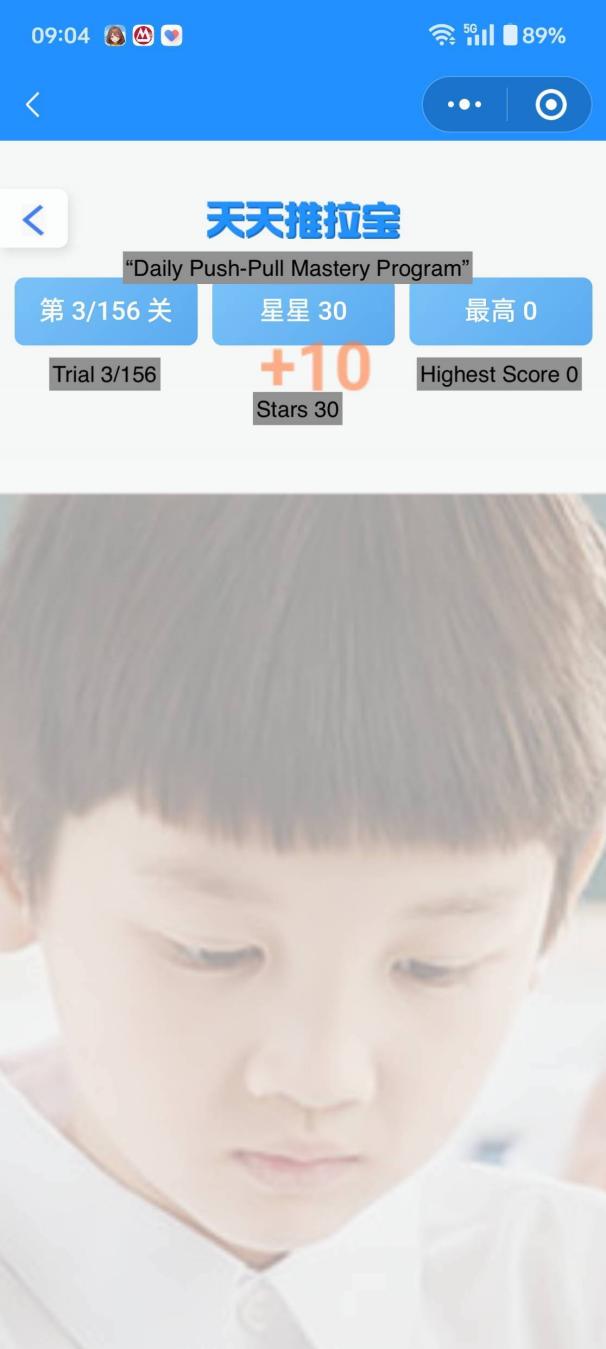

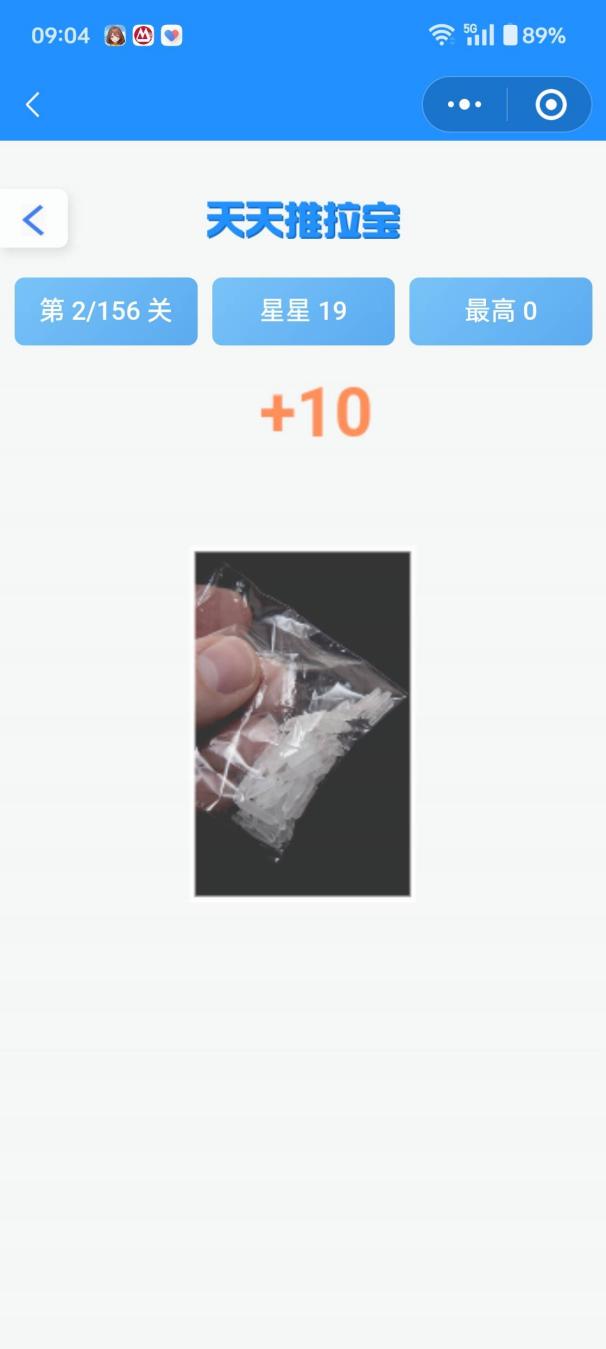

Supplement: Multimedia Appendix 2 [file games-v13-e56978-s002.docx]
